# Multiscale harmonization and semantic integration of biomedical data enable biological insights through immersive exploration

**DOI:** 10.64898/2026.07.07.737090

**Published:** 2026-08-13

**Authors:** Andreas Bueckle, Chenchen Zhu, Alex Yu Hin Wong, Archibald Enninful, Yang Miao, Negin Farzad, Maria Pedersen, Courteney Mattison, Nicholas Sloan, Jason Mares, Cheng Xing, Bruce W. Herr, Juhi Khare, Yash Ramesh Kumar, Keyur Parekh, Siddhi Chavan, Paean Luby, Ushma Patel, Yashvardhan Jain, John W. Hickey, Gary D. Bader, Hemali Phatnani, Vilas Menon, Rong Fan, Peter Sorger, Michael Snyder, Katy Börner

## Abstract

Single-cell atlassing efforts like the Human BioMolecular Atlas Program (HuBMAP) and the Cellular Senescence Network (SenNet) are producing multiscale datasets across the healthy, adult, human body, but this data is typically explored only on 2D screens with limited 3D affordances, even though understanding a cell's location within a tissue, organ, and body requires reasoning across many orders of spatial magnitude. The Human Reference Atlas (HRA) provides standard terminologies and a Common Coordinate Framework (CCF) for harmonizing such data spatially and semantically. Building on the HRA Organ Gallery in virtual reality (VR) application, we present "HRA: Powers of Ten," which integrates, harmonizes, and visualizes biological data from these efforts immersively in VR using a Multiscale Elevator System that lets users descend, like riding an elevator through an inverted skyscraper, from a whole body view of 81 reference organs to datasets across 5 organs (lymph node, brain, large intestine, small intestine, liver), 5 assay types (CODEX, Visium, v-CyCIF, Xenium, SBF-SEM), and 4 spatial scales. Contributed by Data Providers, these VR scenes enable biological insights into senescence patterns, cellular neighborhoods, 3D tissue reconstruction, and subcellular liver architecture. A standard operating procedure supports adding further datasets. Freely available on the Meta Store to over 20 million headset owners, the application, data, and code are open-source.

## Full Text Availability

The license terms selected by the author(s) for this preprint version do not permit archiving in PMC. The full text is available from the preprint server.

